# Dexmedetomidine Improves Postoperative Patient-Controlled Analgesia following Radical Mastectomy

**DOI:** 10.3389/fphar.2017.00250

**Published:** 2017-05-09

**Authors:** Wei Fan, Hong Xue, Yong Sun, HaiKou Yang, Jun Zhang, Guangming Li, Ying Zheng, Yi Liu

**Affiliations:** ^1^Department of Anesthesiology, Huai'an First People's Hospital, Nanjing Medical UniversityHuai'an, China; ^2^Department of Anesthesiology, The Second People's Hospital of Huai'anHuai'an, China; ^3^Department of Burn and Plastic Surgery, Huai'an First People's Hospital, Nanjing Medical UniversityHuai'an, China; ^4^Department of Anesthesiology, Maternal and Child Health Care Hospital of Huai'an CityHuai'an, China

**Keywords:** dexmedetomidine, radical mastectomy, patient-controlled analgesia, morphine

## Abstract

Acute postoperative pain following radical mastectomy is a high risk for prolonged convalescence and potential persistent pain in patients with breast cancer. The present study was designed to observe the effect of intraoperative use of dexmedetomidine on acute postoperative pain following radical mastectomy under general anesthesia. Forty-five patients were enrolled into the study and divided into two groups that were maintained with propofol/remifentanil/Ringer's solution or propofol/remifentanil/Dexmedetomidine followed by morphine-based patient-controlled analgesia. During the first 24 h following surgery, patients receiving dexmedetomine had lower NRS pain scores, decreased morphine consumption, longer time to first morphine request as well as a trending decreased incidence of adverse effects when compared to those received Ringer's solution. In conclusion, the present study finds that intraoperative use of dexmedetomidine could promote analgesic property of postoperative morphine.

## Introduction

A large number of patients underwent radial mastectomy for management of breast cancer experience acute postoperative pain, which leads to prolonged convalescence and additional hospital costs and needs rapid treatment (Jain et al., 2012; Mohamed et al., 2014; Mohta et al., 2016). Postoperative acute pain is also a high risk factor to develop long-lasting chronic hyperalgesia (Jain et al., 2012; Mohta et al., 2016). Morphine is the most currently used analgesic for management of postoperative acute pain (Ge et al., 2015a,b, 2016; Ren et al., 2015; Li et al., 2016; Su et al., 2016; Wang et al., 2016; Zhao et al., 2016). However, morphine induces multiple adverse effects, such as nausea and vomiting, so it is important and urgent to reduce morphine consumption. Aesthesia management may modulate operation-induced postoperative acute pain. Recent clinical trials have found that both intraoperative and postoperative use of dexmedetomidine, a highly selective alpha-2 adrenergic agonist, could facilitate the analgesic property of PCA morphine, reduce morphine consumption as well as its related adverse effects in different types of surgeries, including abdominal surgeries, radical mastectomy, and multi-fracture surgery (Jain et al., 2012; Mohamed et al., 2014; Ge et al., 2015a,b, 2016; Ren et al., 2015; Li et al., 2016; Mohta et al., 2016; Su et al., 2016; Wang et al., 2016; Zhao et al., 2016). Postoperative use of dexmedetomidine was reported to display analgesia-promoting effect on acute and chronic pain in breast cancer patients (Jain et al., 2012; Mohamed et al., 2014; Mohta et al., 2016), however, it is still unknown if intraoperative use of dexmedeomidine has an analgesia-promoting effect on PCA morphine in breast cancer patients undergoing radical mastectomy under general anesthesia. Here, we hypothesized that intraoperative use of dexmedetomidine might promote analgesic effect of PCA morphine, and reduce morphine consumption.

## Results

### Demographic and surgical information of participants

**Figure 1:**
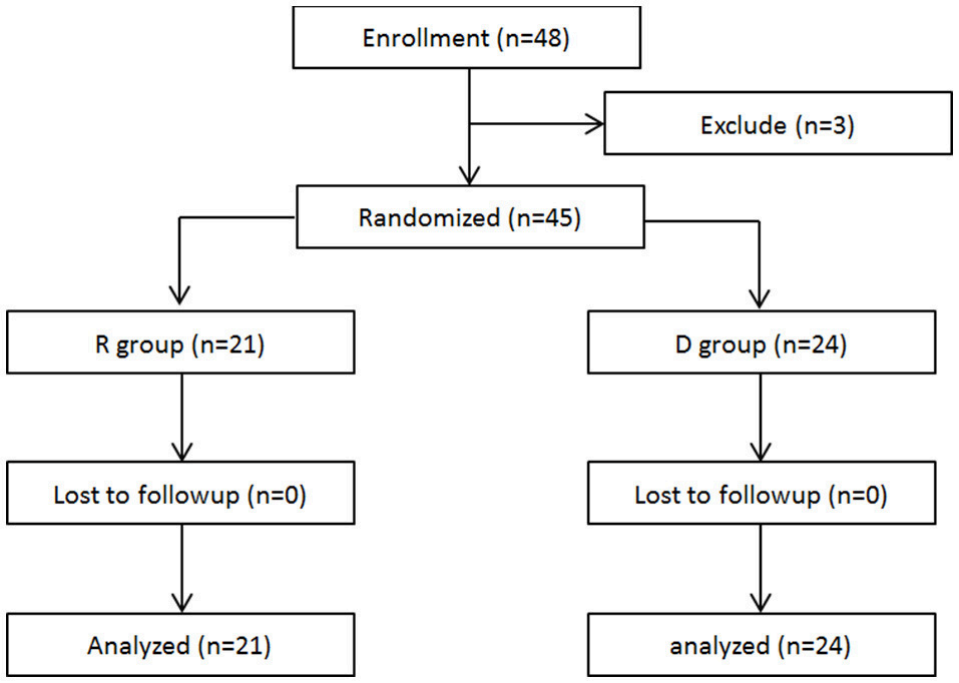
**Flow diagram of the study**.

A total of 48 patients scheduled for radical mastectomy under general anesthesia were assessed for eligibility. Of these, 45 patients were enrolled in this clinical observation trail and randomized into two groups: R group (21 patients) and D group (24 patients), which received either propofol/remifentanil and Ringer's solution or dexmedetomidine (Figure 1). Patients from the two groups were comparable with respect to age, weight, height, BMI, ASA class, operation time, anesthesia time, and PACU time (Table 1).

**Table 1:** **Basic demographic data and surgery duration (mean ± sem)**.

**Variables**	**BS group (*n* = 35)**	**BD group (*n* = 40)**	**Values of *P***
Age (years)	44.29 ± 2.021	43.79 ± 1.809	0.8559
Weight (Kg)	56.82 ± 2.477	56.99 ± 2.019	0.9568
Height	160.3 ± 1.516	161.0 ± 1.424	0.7571
BMI (Kg/m2)	26.87 ± 0.604	26.73 ± 0.4694	0.2648
ASA II/III	17/4	21/3	0.6886
Operation time (min)	131.8.6 ± 3.477	136.4 ± 3.684	0.3721
Anesthesia time (min)	164.6 ± 5.766	169.4 ± 4.745	0.5240
PACU time (min)	42.05 ± 0.8577	45.2 ± 1.325	0.0447

**Figure 2:**
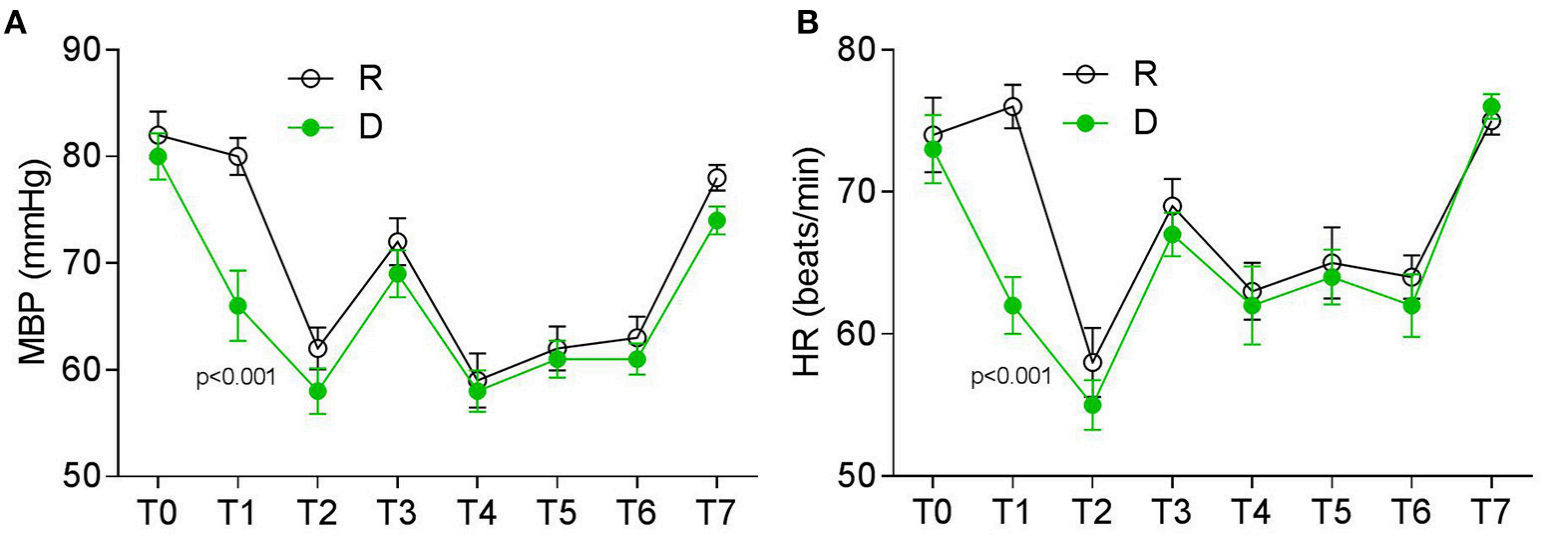
**Perioperative HR and MBP at different timepoints. (A)** MBP (mmHg) and **(B)** HR (beats/min). T0, baseline; T1, time after loading dose; T2, induction; T3, intubation; T4–T6, 30, 60, and 90 min after intubation; T7, 24 h after surgery.

The two groups were also comparable with respect to their baseline mean blood pressure (MBP) and heart rates (HR) before surgery (Figures 2A,B). Patients from the D group experienced a decreased of MBP and HR following a loading dose of dexmedetomidine when compared with those from the R group (Figures 2A,B). All of the patients had a stable but relatively lower MBP and HR than the baseline level during operation, which returned to the baseline levels 24 h after operation (Figures 2A,B).

### Postoperative patient-controlled analgesia of the two groups

**Figure 3:**
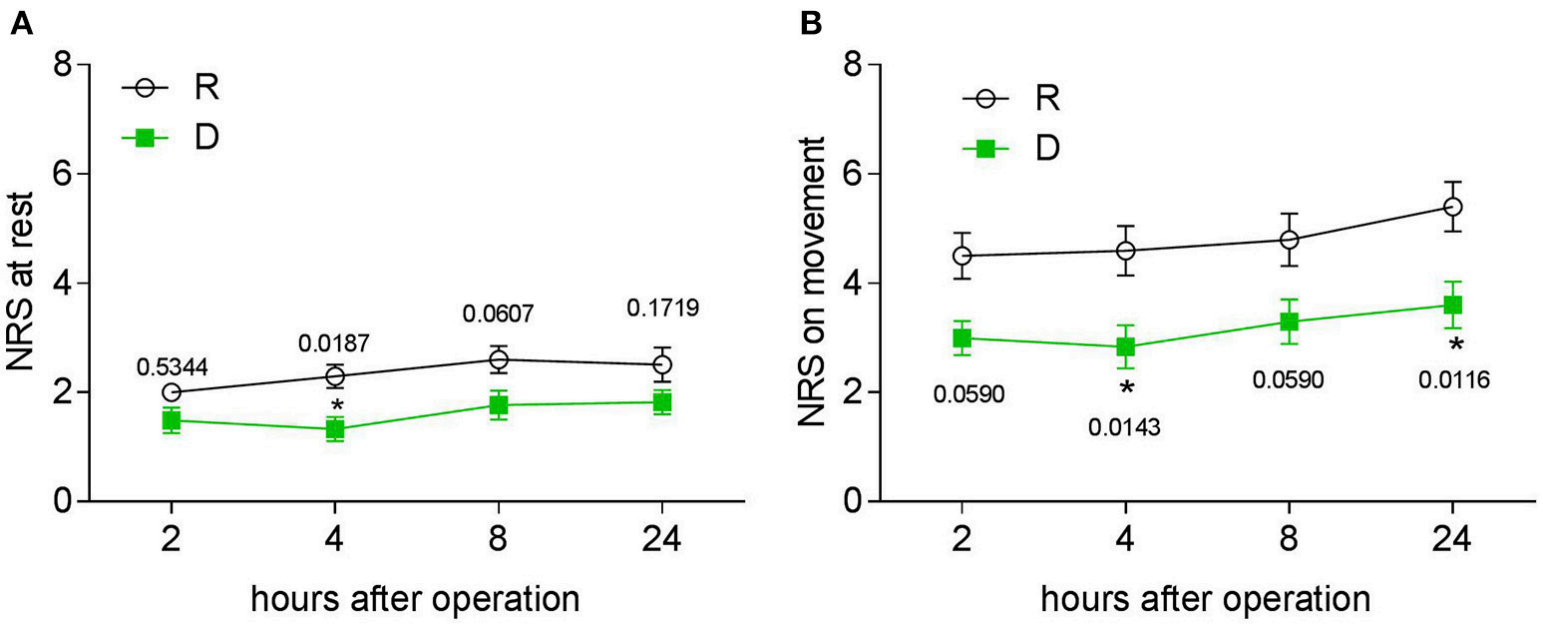
**Twenty-four hour postoperative PCA evaluation. (A)** NRS pain score at rest at different time points in the two groups. **(B)** NRS pain score after coughing at different time points in the two groups, ^*^P < 0.05.

**Figure 4:**
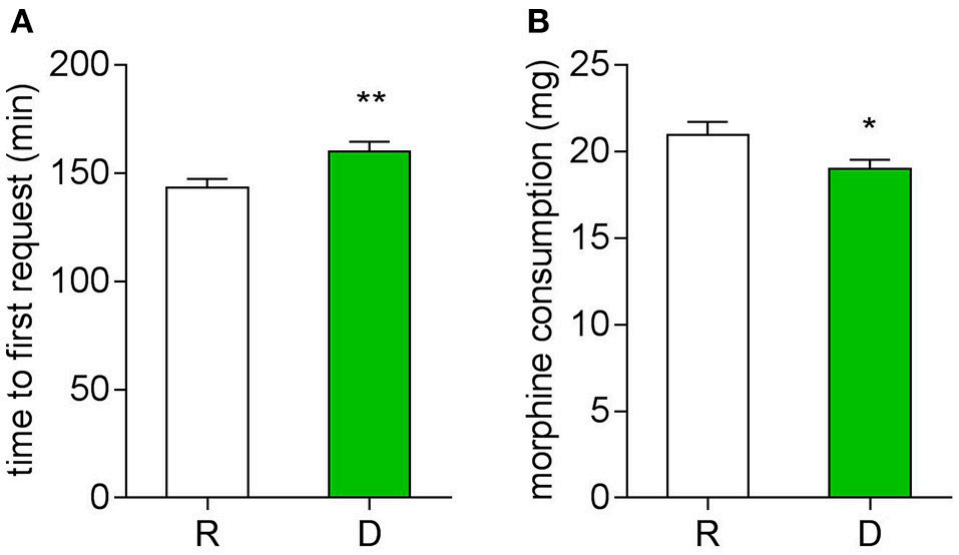
**Twenty-four hour postoperative morphine consumption**. **(A)** Time to first press of PCA pump (^**^P < 0.01). **(B)** Total morphine consumption of the first 24 h (^*^P < 0.05).

Postoperative pain was examined with a NRS, first morphine request time and morphine consumption were noted. During the first 24 h, patients from the D group had a lower NRS score at both the resting (Figure 3A) and movement (Figure 3B) statement, they also had a longer first request time for morphine (Figure 4A) and consumed less morphine (Figure 4B) as compared with patients from the R group.

### Postoperative adverse effects

We detected a decreased incidence vomiting and a trending decrease of nausea during the first 24 h in the patients from D group: less patients of the D groups experienced vomiting and nausea (Table 2). No difference was observed in other adverse effects between the two groups.

**Table 2:** **Adverse effects**.

**Variables (+/−)**	**BS groups**	**BD groups**	**Values of *P***
Nausea	11/10	7/17	0.3499
Vomiting	10/11	5/18	0.0727
Bradycardia	2/19	3/21	>0.9999
Respiratory depression	0/21	0/24	>0.9999
Itching	2/19	2/23	>0.9999

*+/−, positive patient numbe/negative patient number*.

## Discussion

The present study has demonstrated that a combination of general anesthesia with dexmedetomidine promotes analgesic effect of morphine, has a morphine-sparing effect and potentially reduces postoperative nausea and vomiting.

It is widely known that breast cancer patients undergoing radical mastectomy experience severe acute postoperative pain, which might lead to chronic pain (Jain et al., 2012; Mohamed et al., 2014; Mohta et al., 2016). Opioids, especially morphine-based patient controlled analgesia were widely used for postoperative pain control (Ge et al., 2015a,b, 2016; Ren et al., 2015; Li et al., 2016; Su et al., 2016; Wang et al., 2016; Zhao et al., 2016). In terms of the side effects, such as nausea, vomiting, itch, etc., people have been pursuing new novel drugs or drug combinations to reduce the morphine consumption. Dexmedetomidine is an alpha-2 receptor agonist developed in the 1990s, and it was first introduced into hospital as a sedative in ventilated patient in the intensive care unit (Rosenzweig and Sittambalam, 2015). Unexpectedly, it emerged as an adjuvant of local anesthetics and opioids for local and general analgesia (Gruenbaum et al., 2016; Li et al., 2016; Marhofer and Brummett, 2016). Clinical trails have recently reported that intraoperative use of dexmedetominde with or without a loading dose both promoted morphine's analgesic property and reduce morphine consumption (morphine-sparing effect) in patient-controlled analgesia following different kinds of operations (Zhao et al., 2016). It also had a potential to reduce postoperative nausea and vomiting. Morphine-sparing effect of dexmedetomine have also been well established by other early studies both in adults and pediatric patients (McQueen-Shadfar et al., 2011; Gupta et al., 2013; Jones et al., 2014). Recent clinical studies also reported that dexmedetomidine could be used to control postoperative analgesia and chronic pain in breast cancer patients following radical hamstectomy (Jain et al., 2012; Mohamed et al., 2014; Mohta et al., 2016). Consistently, our present study indicated that, in breast cancer patients, intraoperative use of dexmedetomine with a loading dose displayed similar effect on morphine-based PCA. Thus, we believe that intraoperative dexmedetomidine is potentially be used to promote morphine's analgesic effect following radical mastectomy in breast cancer patients. Collectively, intraoperative use of dexmedetomidine might be a novel but useful way to promote postoperative analgesia.

Dexmedetomidine, unexpectedly with a long-term analgesic effect, has a short plasmatic half-time of 2–2.5 h (Grosu and Lavand'homme, 2010). So far, the mechanisms by which dexmedetomidine shows it long-term analgesic effects have remained unknown. A recent study (Ge et al., 2016) summarized several possible mechanisms underlying the long-term analgesic effect: unlike its the sedation effect, dexmedetomidine uses a different α2AR-dependent downstream mechanism to act as an analgesic; dexmedetomidine prolongs the analgesic effect of other analgesics; α2AR in-dependent mechanisms.

The numerical rating scale and visual analog scale are two commonly- and widely-used scales for pain severity evaluation (Ferreira-Valente et al., 2011), and NRS is validated to be more reliable than VAS by a recent clinical trial, so NRS was performed to evaluate postoperative acute pain in the present study.

Collectively, our study found that general anesthesia combined with dexmedetomidine was useful for promoting morphine's analgesic effects in breast cancer patients following radical mastectomy. Possibly, the conclusion could be generalized in female cancer patients with postoperative acute pain. The single sex of the patients might be a limitation of this study, lack of long-term observation should be another one.

## Methods

### Participants

This study was approved by the Institutional Medical Ethics Committee of Nanjing Medical University, and was in accordance with the approved guidelines. Written informed consent was obtained from each patient. A total of 48 patients scheduled for radical mastectomy under general anesthesia were assessed for eligibility. Finally, forty-five patients were enrolled in the study, and assigned into R (*n* = 21), and D (*n* = 24) based on their treatment. Informed consent was obtained from all subjects. The PRS and PRD patients received propofol, remifentanil, and Ringer's solution or dexmedetomidine for general anesthesia maintenance, respectively. To be blind, the maintenance syringe pump was prepared by a different anesthesiologist. NRS scores were evaluated by a different anesthesiologist. Patients matching the following criteria were included in this study: American Society of Anesthesiologists (ASA) grade II or III; between 30 and 60 years old; weight 45–75 kg; height 145–175 cm. Patients were excluded if they had a history of opioid addiction, long-term alcohol abuse or smoking history, current use of sedative–hypnotic drug(s); obesity (BMI > 30); postoperative nausea and vomiting history; or neuropsychiatric diseases and related treatment history.

### Anesthesia

Before induction, patients from the D group received a fast infusion of 100 ml Ringer solution with or without DEX (1 μg/kg) as a loading dose within 15 min. For induction, patients from both groups received midazolam (0.05 mg/kg), remifentanil (2–5 μg/kg), propofol (1.5–2 mg/kg), and cisatracurium (0.2 mg/kg). Immediately after intubation, the patients were ventilated with an oxygen and air mixture (FiO2 = 0.4) with a PetCO2 of 30–35 mmHg. Intravenous infusion was switched to a maintenance syringe pump at rate of 50–80 μg/kg/min for propofol, 0.15–0.2 μg/kg/min for remifentanil, and 0.4 μg/kg/h for dexmedetomidine. Cisatracurium (0.05 mg/kg) was intermittently used for muscle relaxation. They were awoken and extubated, then transferred to the PACU.

Postoperative patient-controlled analgesia: patients were instructed to the use of the numeric rating scale (NRS; 0, no pain, and 10, most severe pain) and the i.v. PCA pump (50 mg morphine and 8 mg ondansetron in 100 ml saline, every pump press leads to a 2 ml of infusion).

### Data collection

Patient demographic information was collected on admission. Hemodynamic indexes were monitored every 5 min during operation. Postoperative pain at rest and on movement states were evaluated with numberic rating scale at different time points post surgery (all time points see figure legends and table notes). Participants that received rescue morphine in the PACU had the rescue morphine included in the total consumption of postoperative PCA morphine. PCA-related adverse effects after operation were recorded.

### Statistics

All data were presented as mean ± sem. or the exact values, and analyzed with GraphPad Prism 6.0 software. Age, weight, height, operation time, anesthesia time and PACU time, morphine first request time and morphine consumption were compared with *unpaired student's t-*test. HR, MBP, NRS at different time points were compared between the two group with *two-way ANOVA* followed by *Bonferroni post-test*. ASA grade and postoperative adverse effects were analyzed with *Fisher's* test. *P* < 0.05 were considered to be significant.

## Ethics statement

This study was carried out in accordance with the recommendations of the Institutional Medical Ethics Committee of Nanjing Medical University with written informed consent from all subjects. All subjects gave written informed consent in accordance with the Declaration of Helsinki. The protocol was approved by the Institutional Medical Ethics Committee of Nanjing Medical University.

## Author contributions

WF, HX, and YS conceived of this study. WF, HX, HY, YS, JZ, GL, YZ, and YL performed the experiments. WF, HY, and YS analyzed the results and wrote the manuscript. All of the authors reviewed the manuscript.

### Conflict of interest statement

The authors declare that the research was conducted in the absence of any commercial or financial relationships that could be construed as a potential conflict of interest.

## Abbreviations

DEX: dexmedetomidine
PCA: patient-controlled analgesia
VAS: visual analog scale
NRS: numerical rating scale
ASA: American Society of Anesthesiologists
BMI: Body mass index
HR: heart rete
MBP: mean blood pressure
PACU: post-anesthesia care unit.
